# Using a Virtual Community of Practice to Support Stroke Best Practice Implementation: Mixed Methods Evaluation

**DOI:** 10.2196/31827

**Published:** 2022-04-27

**Authors:** Elizabeth Linkewich, Sylvia Quant, Lauren Bechard, Michelle Donald

**Affiliations:** 1 North and East GTA Stroke Network Sunnybrook Health Sciences Centre Toronto, ON Canada; 2 Sunnybrook Research Institute Toronto, ON Canada; 3 Department of Occupational Science and Occupational Therapy Faculty of Medicine University of Toronto Toronto, ON Canada; 4 Addiction Services for York Region Aurora, ON Canada

**Keywords:** stroke, rehabilitation, knowledge translation, implementation, quality improvement, evidence-based practice, evaluation, continuing education, social media, internet, web-based learning, allied health

## Abstract

**Background:**

Successful best practice implementation is influenced by access to peer support and knowledge exchange. The Toronto Stroke Networks Virtual Community of Practice, a secure social media platform, is a knowledge translation tool supporting dissemination and adoption of stroke best practices for interprofessional stroke stakeholders.

**Objective:**

The aim of this study is to evaluate the use of a virtual community of practice (VCoP) in supporting regional stroke care best practice implementation in an urban context.

**Methods:**

A mixed methods approach was used. Qualitative data were collected through focus groups and interviews with stroke care provider members of the VCoP working in acute and rehabilitation settings. Thematic analysis was completed, and the Wenger Value Creation Model and developmental evaluation were used to reflect practice change. Quantitative data were collected and analyzed using website analytics on VCoP use.

**Results:**

A year after implementation, the VCoP had 379 members. Analysis of web analytics data and transcripts from focus groups and interviews conducted with 26 VCoP members indicated that the VCoP provided immediate value in supporting user networking, community activities, and interactions. Skill acquisition and changes in perspective acquired through discussion and project work on the VCoP were valued by members, with potential value for supporting practice change. Learning about new stroke best practices through the VCoP was a starting point for individuals and teams to contemplate change.

**Conclusions:**

These findings suggest that the VCoP supports the early stages of practice change and stroke best practice implementation. Future research should examine how VCoPs can support higher levels of value creation for implementing stroke best practices.

## Introduction

### Background

In Canada, stroke care is guided by the *Canadian Stroke Best Practice Recommendations* [1], a resource for health professionals to bridge the gap between scientific knowledge and clinical practices in stroke care. Best practice guidelines are effective in increasing the performance of care processes identified as best practices and in improving patient outcomes [2]. Although best practices can support the consistent delivery of evidence-based care throughout a health system, the implementation of best practices can be challenging because of environmental barriers including organizational constraints, resource allocation, and social and clinical norms that vary among professions, organizations, and regions [3]. To address the challenge of implementing best practices in diverse contexts, a variety of approaches to tailor programs to meet the needs of regional settings and target groups have been developed [3]. Implementation success is influenced by individual factors such as educational interests and motivation [4]. Social factors, such as communication within a social network, accessibility of local opinion leaders, and availability of knowledge exchange with peers, also influence the success of initiatives aiming to achieve practice change in health care [5-8].

Communities of practice (CoPs) are groups of people “sharing a concern, a set of problems, or a passion about a topic, and who deepen their knowledge and expertise in this area by interacting on an ongoing basis” [9,10]. CoPs are knowledge translation (KT) tools that have the potential to support the implementation of practice change initiatives by addressing the communication and social network factors affecting implementation success. Research on CoPs shows that they can contribute to best practice implementation in a variety of health care contexts, including stroke care [11-14]. Virtual CoPs (VCoPs) are a type of CoP that combines the value of traditional face-to-face CoPs in supporting peer-facilitated learning with the value of social media networks to overcome geographical and temporal boundaries to knowledge sharing and continuing education in health care [15]. VCoPs have typically provided a closed, virtual group environment to share knowledge, address professional isolation, network, foster peer collaboration and mentorship, and improve clinical practice through KT [15].

### Rationale

VCoPs are a potentially valuable tool for supporting the implementation of stroke care best practices, as they provide a mechanism to share knowledge among stakeholders across geographical and organizational boundaries. VCoPs also provide a virtual space for care providers from different disciplines to connect. VCoPs provide stakeholders with a platform to share strategies and outcomes of best practice implementation initiatives in different contexts, allowing them to leverage learning from others to make the process of quality improvement more efficient.

The Toronto Stroke Networks (TSNs) work collaboratively with stakeholders to implement high-quality stroke care and have an established education and KT infrastructure grounded in the *Knowledge to Action* (KTA) Framework [16]. Following TSNs stakeholder meetings with clinicians, researchers, managers, and system leaders across 17 regional acute and rehabilitation organizations, the TSNs established a stroke-specific VCoP to support the system-wide implementation of stroke best practices [17]. VCoP membership is largely constituted by regional stroke care providers in a large urban setting; however, there are also members from outside this region. As an ongoing KT initiative, the TSNs VCoP supports stroke best practice implementation by providing discussion forums, organizing working groups, housing a directory of local stroke care stakeholders, and curating a central repository of stroke care resources. In addition to housing passive resources (eg, educational and implementation resources for stroke best practices and members directory), the VCoP was designed to support active engagement and interaction among members through discussion groups and forums. Members may join any open public groups based on specific topics (eg, mood and cognition), in which multiple discussion forums may be housed about different subtopics (eg, depression screening as a subtopic of mood and cognition). Members have the capability to start new discussion threads within a forum or respond to existing threads by posting. Posts can include plain text responses, links, or uploaded resource documents. Documents can also be uploaded to groups as a general resource not associated with a specific post. Members may also request the creation of private groups, which have the same functionality as public groups, but require members to submit a request to join them.

The aim of this study is to evaluate the use of the TSNs VCoP by members and the value created in supporting regional dissemination and implementation of stroke care best practices using a mixed methods approach. This study adds to the small existing body of literature on the use and value of VCoPs to support best practice implementation in health care contexts.

## Methods

### Overview

A mixed methods approach was used in this study to evaluate the TSNs VCoP using both quantitative data on site use and qualitative data on value created through the use of the VCoP. Developmental evaluation [18,19] and the Wenger Value Creation Model [10] were used as foundations to inform the development of the evaluation framework for the VCoP.

### Evaluation Framework

Developmental evaluation seeks to enhance the “understanding [of] the activities of a program operating in dynamic, novel environments with complex interactions” [18] by asking evaluative questions and gathering data to inform ongoing decision-making. Developmental evaluation poses a useful approach for assessing the value and impact of CoPs for supporting best practice implementation because of the complex, social nature of CoPs as an educational strategy. For VCoPs, developmental evaluation is an appropriate approach for assessing the challenge of tailoring implementation for varying contexts identified by individual VCoP members, teams, organizations, and the region.

Although developmental evaluation provides a structured method of assessment, the Wenger Value Creation Model [9] provides a complementary conceptual framework for contextualizing the results of this evaluation. The Wenger Value Creation Model [9] consists of five cycles: cycle 1, *Immediate Value—Activities and Interactions*; cycle 2, *Potential Value—Knowledge Capital*; cycle 3, *Applied Value—Changes in Practice*; cycle 4, *Realized Value—Performance Improvement*; and cycle 5, *Reframing Value—Redefining Success*. The Wenger Value Creation Model [9] supports the evaluation of CoPs by assigning value to the processes and outputs of a network that result in learning enabled by community involvement and networking [10]. The use of this model requires the integration of quantitative indicators and qualitative themes to build a picture of how a CoP creates value for its members [10]. To support data analysis, a framework based on developmental evaluation [18,19] and the Wenger Value Creation Cycles [9,10] was constructed by the authors (MD, EL, and SQ; Table 1).

### Data Collection

Data on VCoP use were collected 1 year after implementation of the VCoP by 2 university students supervised by a member of the TSNs team (JF), including quantitative, aggregate web analytics data (eg, number of members and number of site visits), quantitative user-level data (eg, number of discussion posts), and qualitative user-level data (eg, questions asked in discussion forums and replies to discussion posts). Quantitative data were collected manually from the VCoP and using Google Analytics. Qualitative user-level data were collected manually from the VCoP and did not contain any identifying characteristics of users.

VCoP members were contacted with a request to participate in a focus group or semistructured interview via email and through the VCoP by a member of the TSNs team (JF). Semistructured interviews and focus groups were conducted by a member of the TSNs team (JF) with support from 2 university students following an interview guide with prompts based on the Wenger Value Creation Model [9,10]. Interviews and focus groups were audio recorded and transcribed.

### Data Analysis

To analyze the data collected using web analytics, descriptive analysis of quantitative data on VCoP use was completed using Microsoft Excel (version 2015, Microsoft) to determine the total numbers of page visits, posts, VCoP members, and other indicators of VCoP engagement within 1 year following implementation. Narratives from VCoP discussion forums were analyzed using the analysis framework for this study based on the Wenger Value Creation Model [9,10] (Table 1).

To analyze the data collected from interviews and focus groups with VCoP members, transcripts from audio recordings of interviews and focus groups were generated and analyzed using thematic analysis [20]. Although an interview guide based on the Wenger Value Creation Model [9,10] was used, the analysis was inductive, in that themes developed in the analysis were based on what emerged from discussions in interviews and focus groups, not a predetermined thematic or coding structure.

The analysis was conducted individually by two university students and two authors (MD and EL) with expertise in KT. Manual coding of transcripts was performed to create frameworks by each individual. Subsequently, individual coding frameworks were compared manually to reach a consensus and develop a preliminary coding framework. This framework was then applied in recoding the transcripts. The resulting themes were collectively reviewed for validation through consensus. The trustworthiness of the findings was supported by review and validation of themes by authors who did not participate in the interviews and focus groups (MD and EL). An audit trail of coding changes was created to document the process of analysis and ensure consistency between coders.

Quantitative and qualitative data from web analytics and interviews and focus groups with participants were integrated using the evaluation framework developed for this study (Table 1) to identify broader themes of value creation stories generated from VCoP use.

### Ethics Approval

Ethical review and approval for this study were provided by the Sunnybrook Health Sciences Centre Research Ethics Board before the collection of data (approval number 123-2013). Quantitative data on VCoP use were anonymous, and qualitative user-level data were anonymized and deidentified at the time of collection. Informed consent to participate in interviews and focus groups was provided by VCoP members interested in participating in the study. Any identifying details presented in interviews were omitted from transcripts.

**Table 1:** VCoP^a^ evaluation framework using the Wenger Value Creation Cycles.

Cycle and cycle indicator		Quantitative data (web analytics)	Qualitative data (interviews and narratives from VCoP)
**Cycle 1: Immediate Value—Activities and Interactions**			
	Level of participation	Number of threads, total number of members, number of members in a group, number of members in discussion forums, and number of discussion forums	Questions or statements related to joining a discussion forum
	Quality of interaction	Frequency of responses to inquiries, frequency of citing one’s own experience, post length (words), number of debates or differing points of view, and number of suggestions made to a problem	Questions or statements about a clinical issue and sharing of a case example
	Networking	Number of group memberships	Question or statement about a need that can be met by the expertise of another member and request connection with a member’s particular knowledge
	Collaboration	Number of joint projects and timeliness of responses	Question or statement about a collaborative project and statement about length of time a member waited for response to a question
**Cycle 2: Potential Value—Knowledge Capital**			
	Skills acquired	Number of documents	Question or statement about a document on the VCoP that was used or shared with colleagues
	Change in perspective	—^b^	Statements indicating a shift in understanding or opinion
	Confidence building	—	Questions or statements indicating disagreement or challenging posts of other members
**Cycle 3: Applied Value—Changes in Practice**			
	Implementation of advice, solutions, and insights	—	Questions or statements about successes or challenges related to applying new learning from the VCoP
	Use of social connections	—	Questions or statements about networking with other VCoP members
**Cycle 4: Realized Value—Performance Improvement**			
	Personal performance	—	Statements about personal goals
	Organizational performance	—	Statements about organizational goals or accomplishments
	Organizational reputation	—	Statements about organizational performance relative to other benchmarks (eg, Ministry of Health and Long-Term Care and Health Quality Ontario)
**Cycle 5: Reframing Value—Redefining Success**			
	Community aspirations	—	Questions or statements that indicate new purpose for the VCoP or improvements and/or additions to the community
	Relationships with stakeholders	—	Questions or statements about relationship building or with others external to the VCoP (eg, patients or other organizations)

^a^VCoP: virtual community of practice.

^b^No quantitative data identified.

## Results

### Overview

A year after implementation, 379 members had joined the VCoP from 22 organizations in the TSNs representing several professional backgrounds: nursing, occupational therapy, physical therapy, speech-language pathology, medicine, academic research, and other health system stakeholders. Of these 379 members, 26 (6.9%) provided informed consent and participated in 14 interviews and 2 focus groups. Participants included nurses, occupational therapists (OTs), physicians, physiotherapists (PTs), and speech-language pathologists (S-LPs) from 4 rehabilitation and 8 acute care organizations. Overall, 19 participants provided data for number of years in practice, with the mean number of years practicing being 15.6 (SD 8.7; range 2-29.5) years.

### Quantitative Results

The data collected from the VCoP through web analytics reflecting cycles 1 and 2 of the evaluation framework are summarized in Table 2.

**Table 2:** Quantitative data summary.

			Value
**Descriptive web analytics**			
	Virtual community of practice members, n	379	
	Groups, n	24	
	Discussion forums, n	21	
**Discussion forum descriptive analytics**			
	Threads initiated in each discussion forum, mean (SD)	1.04 (1.4)	
	Discussion threads in each group, mean (SD)	1.0 (1.4)	
	Posts in each thread, mean (SD)	2.5 (5.1)	
	Prompts and questions per discussion forum, n	28	
	Responses to inquiries, n	27	
	Instances own experience cited, n	6	
	Debates about a topic, n	0	
	Length of posts (number of words), mean (SD)	67.2 (65.1)	
	Members in each group, mean (SD)	17.0 (41.2)	
	Members in a discussion forum, mean (SD)	1.9 (1.7)	
**Member behavior descriptive analytics**			
	Documents shared, n	117	
	Suggestions made to a problem, n	31	
	Joint projects, n	9	
	Timeliness of responses (number of days), mean (SD)	18.6 (14.2)	

### Qualitative Themes

The subjective value of the VCoP and use patterns were collected using interviews and focus groups with VCoP members. Through a qualitative thematic analysis of interview and focus group transcripts, the research team identified 5 themes discussed in the next sections.

#### Theme 1: Effective Networking

Participants noted that a primary function of the VCoP is to provide a more effective platform for contacting individuals and building networks within and across disciplines. This enhanced connectivity was primarily achieved through the use of the VCoP as a mechanism to find members’ organizational contact information and directly message them through this platform. Participants noted that they leveraged the VCoP network to assist colleagues who were not VCoP members, suggesting the spread of VCoP value beyond the immediate member network:

...I helped a colleague here who needed to contact somebody...and you know, through the VCoP I knew exactly who she needed to contact. I got her number through the member directory.

Participant 17, OT

What is meaningful to me is directly messaging other members, I can network with people in the same discipline.

Participant 2, S-LP

#### Theme 2: Value for Project-Based Work

Participants also discussed how they leveraged the VCoP to support collaborations within and between professions on ongoing stroke projects. Members also used the VCoP to collaborate with individuals on posters for conferences, which contributed to knowledge transfer outside the VCoP:

We’ve been using [the] VCoP for the development of posters...to share ideas & information about the process, and then post the actual creation of posters on VCoP...that was our only means of communication at that point...I was able to liaise with different [colleagues] about different practice concerns...the two posters I worked on.

Participant 10, OT

This project-based collaboration on the VCoP was noted to provide additional educational opportunities. For example, individuals not directly involved in he projects benefited from observing the collaborative process in public groups, leading to the development of conference posters. In addition, VCoP members identified that conducting project-based work on the VCoP led to the development of practice resources:

I have learned things from reviewing the other posters that I haven’t been involved in because I was part of the online group and saw what was posted.

Participant 6, nurse

[The] VCoP was the means of communication for a group project...afterwards, I created additional assessment checklists...and shared it with my team.

Participant 19, OT

#### Theme 3: Resource Sharing Supports Application of Knowledge to Practice

Participants identified that access to up-to-date information and resource sharing improved with VCoP use. The VCoP was noted as beneficial for identifying topic-specific resources more easily and providing a central location for resources. This increased accessibility saved members’ time that would otherwise have been spent searching for these resources:

Now with the VCOP, I will not have to go back to my desk to refer to the assessment checklist as it is all online now.

Participant 16, nurse

I printed the triage tool that someone uploaded to the VCoP and gave it to new people on my team for reference.

Participant 17, PT

VCoP members also noted that these resources had an impact beyond their immediate use. Resource sharing on the VCoP provided a catalyst for in-person knowledge sharing about best practices across institutions. Although a direct link to changes in practice was not identified, participants noted that VCoP resources started discussion about changes in practice that could lead to future changes:

I shared information with a group that sparked discussion about [specialized] assessments, the comparison between hospitals prompted discussions here with our [professional group] about whether we should change our ways of doing things.

Participant 13, OT

#### Theme 4: Enhancing Understanding of Stroke Best Practices and Stroke Care Priorities

Participants identified that the VCoP helped them understand the broader picture of stroke best practices within their region beyond their disciplinary boundaries. Participants noted that the VCoP helped them access information to support learning about stroke Quality-Based Procedures (QBPs) [21], which are stroke care procedures associated with improved outcomes and reduced financial cost to the health care system:

Looking up the information about the QBP is very helpful to get someone new to understand how it impacts the [stroke] program...it gives them a leverage point of how they can advocate for the stroke patients.

Participant 17, PT

Multiple participants noted that the information shared on the VCoP went beyond what was available from other web-based sources and was more regionally relevant. Learning about activities and concerns outside one’s practice was made possible through a diverse membership on the VCoP from across professions, sectors, and organizations within the region:

So if you really want to find out what’s happening across the stroke community, you have that opportunity more at your fingertips than what you did before.

Participant 8, PT

It’s information that you’re not going to necessarily find on the internet because it’s about current practice.

Participant 12, OT

I have a better awareness of the best practices and what the bigger picture is. When you work in one area (e.g. rehab) you don’t really have a big sense of what was going on in acute care, what they were looking at for outpatient services, community service...being a part of the community and through looking at some of the resources.

Participant 8, PT

#### Theme 5: Barriers to Use

Although themes of benefits were identified, challenges with accessing resources were also recognized as barriers to VCoP use. Members noted that the navigation path to certain resources was too long, making them difficult to find. Frustration over inability to find resources and time spent searching for them was noted as a deterrent to future use:

If it takes less time to find it, right, it’s like you’re spending like 10 minutes and you’re like, “Okay, I’m not going to do this again,” right?

Participant 2, S-LP

Email notifications were suggested as a strategy to increase engagement in preferred topics and previous discussions in which individuals had participated. This suggested that improvement may decrease the time spent searching the site for information and challenges with navigation:

...if there was a new tag or a new thing, something pops up into my e-mail, to, kind of, go answer it...what about an option to receive updates via email, or notifications based on individuals’ interests?

Participant 9, physician

### Integrating Quantitative Data and Qualitative Themes

**Figure 1:**
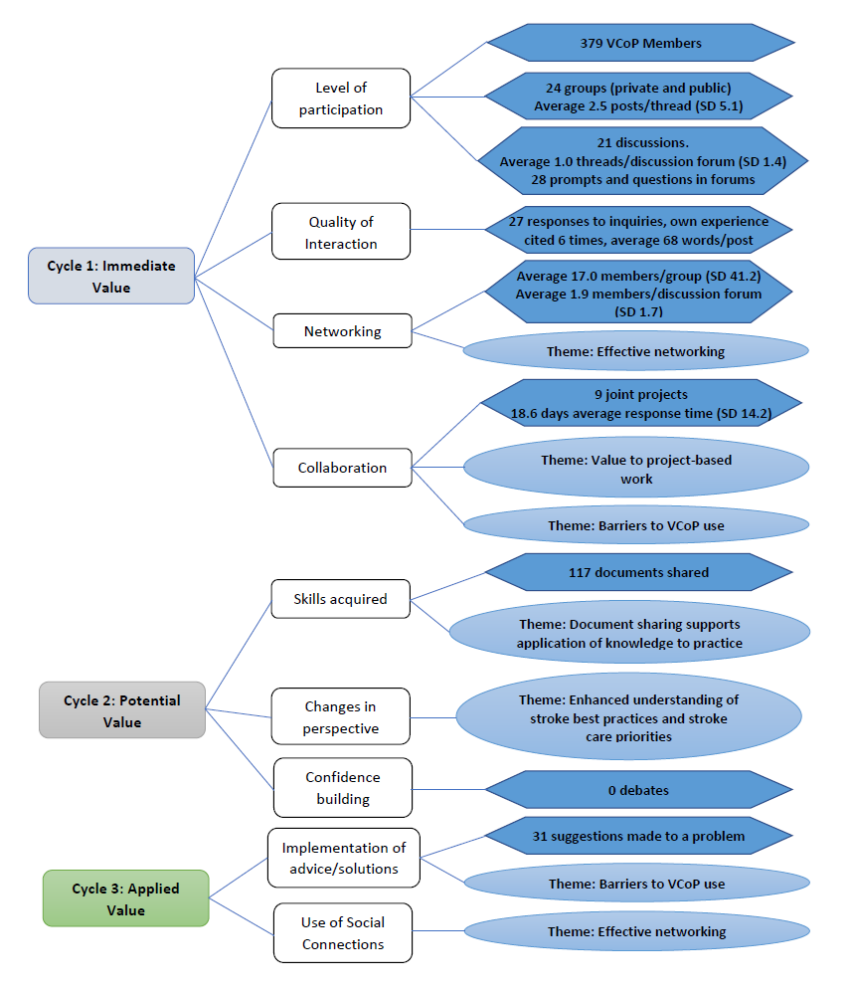
Mapping of quantitative data and qualitative themes to an evaluation framework reflecting the Wenger Value Creation Model (cycles 1-3). VCoP: virtual community of practice.

The results from both quantitative and qualitative analyses were applied to the evaluation framework. These results provide evidence for value creation associated with VCoP reflective of cycles 1 to 3 of the Wenger Value Creation Model (Immediate Value, Potential Value, and Applied Value, respectively; Figure 1).

## Discussion

### Principal Findings

The results of this evaluation strongly support the creation of *Immediate Value—Activities and Interactions* (cycle 1) and *Potential Value—Knowledge Capital* (cycle 2), with some evidence provided for creation of value in *Applied Value* (cycle 3) in a VCoP to support stroke best practice implementation [9]. Support for the creation of immediate value in activities and interactions is evidenced by value creation stories identified in this study that described networking, collaboration, and acquiring skills and perspectives through document sharing as meaningful forms of VCoP use for members. Several groups and discussion forums were created during the evaluation period, providing a platform for networking and collaboration. Evidence supporting the creation of value in knowledge capital was evidenced by the number of resources shared by VCoP members during the evaluation period and participants’ narratives about using resources from the VCoP to initiate conversations with their teams about best practice change. Participants frequently cited this as a starting point for team discussions and questioning of current practice that could lead to practice change. The number of responses to inquiries and suggestions made by members demonstrates the responsiveness of the community to one another.

Although evidence for value creation reflective of cycle 3 was weaker than that of cycles 1 and 2 in this study, some narratives and data on the number of projects, number of resources shared, and number of solutions to problems offered by members support the initial steps toward best practice implementation. Several VCoP member narratives described sharing of resources as helping individuals and teams make progress toward changes in practice. For example, VCoP members interacting in a group (cycle 1) supported the generation of knowledge capital such as an educational poster (cycle 2). In the process, members learned from each other and brought information back to their teams, prompting discussions about practice changes (cycle 3).

Evidence of the value created by a VCoP to support stroke best practice implementation in this study aligns with existing KT frameworks that identify the importance of increasing awareness of best practices and identifying practice gaps. For example, the KTA Framework [16] outlines the importance of becoming aware of best practices before determining the gaps, which, in this study, was achieved through interactions between VCoP members and collaboration to create new knowledge products addressing identified gaps. In another framework, Pronovost et al [22] described a collaborative model for integrating theory into practice that engages staff in understanding the current best practice gap and the potential negative consequences of the gap. The KTA Framework [16] and the Wenger Value Creation Model [9] both identify the need to build awareness and prompt collaborative thinking about practice gaps as foundations for successful implementation, which aligns with what was observed in this evaluation.

This evaluation found few instances of members sharing their experiences or debating issues, suggesting that this community is still in the process of building social capital. Social capital is created in social networks [23] through relationship formation and is supported by mutual confidence and trust [24,25]. Members may be hesitant to challenge others or share experiences that could be perceived as organizational or personal shortcomings in a web-based forum such as a VCoP. Previous research has suggested that developing social capital requires more time [26]. This may also explain why this study found evidence for the creation of value in cycles 1 to 3 but less evidence of value created in cycles 4 and 5. It is possible that a data collection period of 1 year may have been too brief, for this growing community, to capture VCoP value in cycles 4 and 5. As the VCoP builds and becomes more deeply integrated into the TSNs’ initiatives, more evidence for cycles 4 and 5 may emerge. Research should continue to evaluate mature VCoPs, as this technology is increasingly being adopted to support KT and interprofessional collaboration.

The patterns of and reflections on VCoP use identified in this study may pose challenges to value creation on the VCoP. Web analytics data indicated slow response times for users to address questions posed by others on the VCoP and instances of as few as 2 individuals having web-based exchanges in some discussion forums, whereas other forums had extensive resource sharing. Slower response times and inconsistent engagement across different discussion forums could challenge value creation on the VCoP by discouraging members from posing questions if they do not think they will receive a timely response or any response at all. Although some VCoP members identified that the VCoP saved them time in the long run when it came to finding resources, others, as supported by previous literature [15,27], also identified the amount of time required to navigate the VCoP as a potential challenge to use. A shorter click path required to access sections of the site, active facilitation of the VCoP (eg, prompting responses and connecting members to answer each other’s questions), and more notifications for personally relevant site activity, as suggested by participants, may lead to a greater number of responses and sharing of experiences.

The self-selection of VCoP members to participate in the focus groups and interviews, rather than random sampling, creates a potential sampling bias that could impact the generalizability or transferability of this work to different regional contexts or KT applications. Additional insights provided by less active members could improve understanding of VCoP value, as active members outweighed nonactive members participating in focus groups and interviews.

Participants included health care providers in acute and rehabilitation hospital settings, which represents VCoP membership demographics at the time of data collection. Membership has since expanded to include more VCoP members from nonhospital settings. Although the VCoP has an interprofessional membership, most participants in focus groups and interviews were allied health professionals. A more diverse group of participants may affect the value expressed by VCoP members for health professions involved in stroke care.

In addition, this study was conducted to evaluate a regional best practice implementation tool. Study participation included users in a large metropolitan area. Although there are existing face-to-face opportunities to build social capital in this region, challenges for stroke care providers to access these opportunities exist (eg, taking time away from care provision to commute to in-person events) [28]. This feedback from stakeholders prompted the development of the TSNs VCoP as a virtual KT resource. Previous research has shown that VCoPs are an effective tool for addressing geographical boundaries to collaboration among health care providers [15]. Some studies have suggested that individuals in more rural areas, where stroke teams are more isolated, may be more motivated to engage in alternate ways to learn and decrease isolation [29,30]. This virtual KT resource was developed to address geographical challenges to in-person engagement in an urban setting; however, they are potentially relevant to both urban and rural settings. Future research should compare the value of VCoPs as a tool for KT in different regional contexts.

### Conclusions

Through a developmental evaluation approach, the value of a VCoP in supporting the initial phases of stroke best practice implementation was demonstrated in this study. Members reported enhanced networking, more efficient identification of resources, and enhanced collaboration on joint projects to build and maintain professional relationships. VCoP use contributed to a broader understanding of the stroke continuum of care and a better understanding of the priorities in stroke care that supported individuals and teams to contemplate best practice change. Future VCoP design changes to improve functionality and user experience were suggested to increase collaboration and the quality of engagement. The evaluation framework used in this study will continue to be used to collect evidence of the value created by the TSNs VCoP as an ongoing KT initiative. The use of a VCoP in other care networks and regions should be investigated to gauge the potential value of this educational strategy for health professionals working with other populations seeking to enhance best practice implementation.

## Abbreviations

CoP: community of practice
KT: knowledge translation
KTA: Knowledge to Action
OT: occupational therapist
PT: physiotherapist
QBP: Quality-Based Procedure
S-LP: speech-language pathologist
TSN: Toronto Stroke Network
VCoP: virtual community of practice
